# Yoga on Our Minds: A Systematic Review of Yoga for Neuropsychiatric Disorders

**DOI:** 10.3389/fpsyt.2012.00117

**Published:** 2013-01-25

**Authors:** Meera Balasubramaniam, Shirley Telles, P. Murali Doraiswamy

**Affiliations:** ^1^Department of Psychiatry and Behavioral Sciences, Duke University School of MedicineDurham, NC, USA; ^2^Indian Council of Medical Research Center for Advanced Research in Yoga and Patanjali Research FoundationBengaluru, India; ^3^Duke Institute for Brain SciencesDurham, NC, USA

**Keywords:** yoga, meditation, depression, schizophrenia, cognition, ADHD, clinical trials, alternative medicine

## Abstract

**Background:** The demand for clinically efficacious, safe, patient acceptable, and cost-effective forms of treatment for mental illness is growing. Several studies have demonstrated benefit from yoga in specific psychiatric symptoms and a general sense of well-being.

**Objective:** To systematically examine the evidence for efficacy of yoga in the treatment of selected major psychiatric disorders.

**Methods:** Electronic searches of The Cochrane Central Register of Controlled Trials and the standard bibliographic databases, MEDLINE, EMBASE, and PsycINFO, were performed through April 2011 and an updated in June 2011 using the keywords yoga AND psychiatry OR depression OR anxiety OR schizophrenia OR cognition OR memory OR attention AND randomized controlled trial (RCT). Studies with yoga as the independent variable and one of the above mentioned terms as the dependent variable were included and exclusion criteria were applied.

**Results:** The search yielded a total of 124 trials, of which 16 met rigorous criteria for the final review. Grade B evidence supporting a potential acute benefit for yoga exists in depression (four RCTs), as an adjunct to pharmacotherapy in schizophrenia (three RCTs), in children with ADHD (two RCTs), and Grade C evidence in sleep complaints (three RCTs). RCTs in cognitive disorders and eating disorders yielded conflicting results. No studies looked at primary prevention, relapse prevention, or comparative effectiveness versus pharmacotherapy.

**Conclusion:** There is emerging evidence from randomized trials to support popular beliefs about yoga for depression, sleep disorders, and as an augmentation therapy. Limitations of literature include inability to do double-blind studies, multiplicity of comparisons within small studies, and lack of replication. Biomarker and neuroimaging studies, those comparing yoga with standard pharmaco- and psychotherapies, and studies of long-term efficacy are needed to fully translate the promise of yoga for enhancing mental health.

## Background

Mental illnesses are asignificant global health concern, despite improvements in treatment modalities and access to care. The World Health Organization (WHO, 2011) has estimated that psychiatric disorders are the leading costs of disability adjusted life years world-wide, with recent figures indicating that 37% of the loss of healthy years from non-communicable diseases is from mental illnesses. The National Co-morbidity survey replication conducted in the United States estimated the 1-year prevalence of any psychiatric disorder to be 26.2% (Kessler et al., 2008). According to the WHO, depression ranked third among global disease burdens all over the world in 2004; it was reportedly the most important cause in middle and high income countries, while it ranked eight among the low income countries (World Health Organization, 2008). Depression was found to result in the greatest decrement in health, compared to asthma, angina, arthritis, and diabetes (Maussavi et al., 2007). Prevalence data for anxiety disorders, suggests that the lifetime prevalence and 12 month prevalence for any anxiety disorder are over 15 and 10%, respectively, with higher prevalence in developed countries (Kessler et al., 2009). Likewise, schizophrenia has been associated with significantly higher health care costs, unemployment rate, and morbidity (Goeree et al., 2005). Sleep complaints are often associated with a variety of psychiatric disorders. About 9–21% of the population has been estimated to have insomnia accompanied by serious day-time consequences which include chronic fatigue, irritability, low mood, memory impairments, and interpersonal difficulties (Moul et al., 2002). This problem has reached epidemic proportions in the United States, where almost 25% of adults consume sleep medications at some point in a year (National Sleep Foundation, 2005).

The availability of psychopharmacological treatments has increased, but the response and tolerability remain unpredictable and inconsistent. While psychotropics agents can be lifesaving for many people, there remains a considerable unmet need. The landmark National Institute of Mental health (NIMH) funded Sequenced Treatment Alternatives to Relieve Depression (STAR*D) study showed remission in only one third of major depression patients after a trial with the first anti-depressant and worsening response rates with each subsequent trial (Trivedi et al., 2006). The Primary Care study conducted by WHO found that 60% of the patients continued to meet criteria for depression after a year of being treated with an anti-depressant (Goldberg et al., 1998). The Clinical Anti-psychotic Trials of Intervention Effectiveness (CATIE) demonstrated that 74% of the participants discontinued from their treatments in 18 months, with a mean time to discontinuation of 4.6 months (Lieberman et al., 2005). Treatment resistance is a growing problem and there are millions of patients world-wide whose depression, anxiety, or schizophrenia is not fully resolved despite multiple trials of psychopharmacologic agents. Psychotropic medications are costly and suffer from significant side effects leaving patients and clinicians to struggle to balance efficacy against cost and side effects, which often leads to poor compliance and relapse.

Given the heterogeneous nature of psychiatric conditions, with respect to biological, psychological, and social factors, it is not surprising that available standard treatments often have inconsistent response rates. The quest and demand for non-pharmacological treatment modalities has been increasing (Barrows and Jacobs, 2002). A study conducted by the Harris Interactive Service Bureau revealed that 15.8 million adults in the United States practice yoga, triple the number in 2004. The holistic goal of yoga to promote physical and mental health, and also be spiritually and socially conscious, may appeal both to consumers and providers who are concerned about the symptom reduction based focus of psychopharmacology and finding inner peace (Uebelacker et al., 2010). The barriers to access are low and the diversity of practice styles and settings (e.g., at home versus in gyms versus outdoors) allows considerable degree of personalization. Hence, yoga appears to be a well suited intervention to test as a potential therapy for major psychiatric disorders. However, yoga has also become such a cultural phenomenon that it has become difficult for physicians and consumers to differentiate legitimate claims from hype. Our goal in this review was to examine whether the evidence matched the promise.

**Figure 1:**
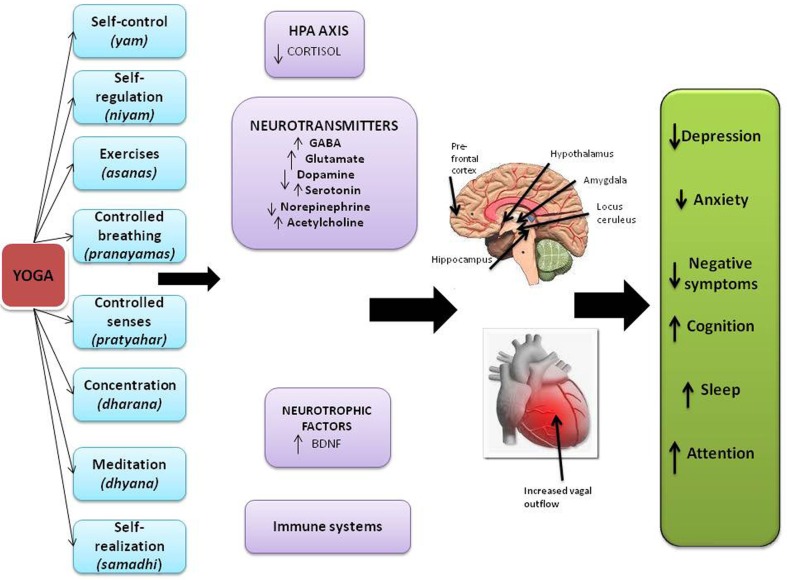
**Schematic illustration of potential effects of yoga on biomarkers and end organs based on various sources**. The strength of evidence ranges from strong to preliminary for specific effects as described further in the text. Copyright Doraiswamy and Balasubramaniam, reproduced with permission in this article.

Yoga, with origins in ancient India has several sub-types (Table 1; Cook, n.d.), and incorporates physical postures (asanas), controlled breathing (pranayama), deep relaxation, and meditation (Javnbakht et al., 2009). In addition to low barriers to access, the scientific rationale for yoga effects on the mind are quite strong. All yoga practices are known to influence the mental state (Telles, 2010) – studies have noted benefits in children (Manjunath and Telles, 2004), adults (Vialatte et al., 2008), elderly (Krishnamurthy and Telles, 2007), and individuals with occupational stress (Vempati and Telles, 2000). In healthy individuals, biomarker studies suggest that yoga influences neurotransmitters, inflammation, oxidative stress, lipids, growth factors, and second messengers (Figure 1), in a manner largely similar to what has been shown for anti-depressants and psychotherapy. It is hypothesized that yoga combines the effects of physical postures, which have been independently associated with mood changes (Phillips et al., 2003), and meditation which increases the levels of Brain-derived neurotrophic factor (BDNF; Xiong and Doraiswamy, 2009). Other effects that have been noted include increased vagal tone, increased gamma-aminobutyric acid (GABA) levels, increase in serum prolactin, downregulation of the hypothalamic-pituitary-adrenal axis and decrease in serum cortisol, and promotion of frontal electroencephalogram (EEG) alpha wave activity which improves relaxation (Janakiramaiah et al., 1998, 2000; Kamei et al., 2000; Streeter et al., 2007). Lastly, prior clinical studies have noted several psychiatric conditions for which yoga has proved beneficial (Shannahoff-Khalsa et al., 1999; Carei et al., 2010; Visceglia and Lewis, 2011; Katzman et al., 2012; Libby et al., 2012) but because of differing methods there is a need to try to synthesize such data to further the field.

**Table 1:** **Table showing the key elements of the different forms of yoga (Cook, n.d.)**.

Type of yoga	Key features
Ashtanga yoga	Fast-paced series of sequential posture, based on six series of asanas
Hatha yoga	Basic form of yoga which incorporates postures, regulated breathing, and meditation
Iyengar yoga	Focuses on the precise alignment of postures
Power yoga	Westernization of Ashtanga yoga. Popular in the US
Jivamukti yoga	Physically challenging postures, highly meditative
Kali Ray TriYoga	Consists of flowing, dance-like movements, often accompanied by music
White Lotus Yoga	Consists of flowing movements with varying difficulty levels
Integrated yoga therapy	Designed for medical problems. May include meditation and guided imagery
Viniyoga	Gentle practice which particularly emphasizes on the synchronization of poses with breathing
Svaroopa	Emphasizes on the “opening of the spine beginning at the tailbone progressing through each spinal area”
Bikram Yoga (Hot Yoga)	Consists of a series of 26 postures performed in a space with temperature above 100°F
Phoenix rising yoga therapy	Combines traditional yoga with client centered and mind-body psychology, that incorporates non-directive dialog
Sivananda yoga	Consists of 12 basic yoga postures along with chanting and meditation
Integral yoga	Consists of basic hatha yoga postures
Ananda yoga	Consists of basic hatha yoga postures with use of “silent affirmations while holding up a pose”
Kundalini yoga	Focuses on awakening the energy at the base of the spine and channeling it upwards
ISHTA yoga	Combination of Ashtanga and Iyengar yoga
Kripalu yoga	Consists of three stages namely willful practice, willful surrender, and meditation in motion
Anusara yoga	Consists of basic hatha yoga postures but emphasizes on *attitude*, *alignment*, and *action*
Tibetan yoga	Composed of fine, flowing movements, and controlled breathing

Thus, while the effects of yoga on the spiritual aspects of the mind (e.g., inner peace) are well documented, its effects in major clinical psychiatric disorders are less so. The objective of this report was to systematically review the available literature for the effects of yoga on major psychiatric disorders. The focus of this review was primarily categorical disease threshold outcomes (e.g., major depression), in keeping with how psychiatric disorders are categorized and treated, and how effects of psychopharmacologic interventions are assessed – rather than on single symptom domains such as mood or sleep which cut across multiple diagnoses. We did use symptoms (e.g., depression and memory) as search terms to ensure our search was comprehensive but restricted our final review to major disorders that require intervention in practice.

## Methods

### Search strategy

Electronic searches of The Cochrane Central Register of Controlled Trials (CENTRAL) and the standard bibliographic databases, MEDLINE, EMBASE, and PsycINFO, was conducted through April 2011 and updated in June 2011, using the keywords yoga AND psychiatry OR depression OR anxiety OR schizophrenia OR cognition OR memory OR attention AND randomized controlled trial (RCT). The title and abstract of each citation were screened based on definite pre-specified inclusion and exclusion criteria. Full text reading of articles that were potentially eligible was undertaken. When full-texts were not available, attempts were made to contact the author. If a reply was not received within 2 weeks from the corresponding author, abstracts were read to check if they had the required information. Studies have been reviewed by all authors and disagreements were resolved by consensus.

Randomized clinical trials with any sub-type of yoga as the intervention and one or more of the above mentioned conditions as the outcome of interest were included. Open trials, non-randomized trials, case series, and dissertations were excluded. The review includes studies in which subjects have either been formally diagnosed with a disorder or have reported symptoms suggestive of the same. Since age is an important risk factor for cognitive impairment, studies examining cognition in the geriatric population have been included, even in the absence of formal diagnoses or specific symptoms. Studies on sub-threshold symptoms such as general well-being, stress, and coping have been excluded. Outcomes consisted of self-reported change, scores on rating scales, acceptability, and tolerance of the treatment.

The quality of RCTs was scored using the guidelines recommended by the Agency for Healthcare Research and Quality (AHRQ, 2002), with a maximum possible score of 17. Table 2 illustrates the scoring according to the AHRQ guidelines. Study quality was additionally assessed using the Oxford Center for Evidence-based Medicine’s (CEBM) Levels of Evidence, which assigns a level of evidence from 1 to 4, where 1 indicates high quality RCTs, 2 indicates low quality RCTs, 3 suggests case-control studies, and 4 stands for case reports, case series, and low quality case-control studies (Phillips et al., 2001). Determinants of study quality have been explained in Table 3. Based on evidence levels obtained by the Oxford CEBM method, recommendation categories of A (recommended), B (suggested), or C (may be considered) have been specified for each diagnosis, as indicated by the Research and Development/University of California at Los Angeles (RAND/UCLA) Appropriateness Method (Fitch et al., 2000; Table 4). In the tables demonstrating details of individual studies for each diagnosis, the AHRQ scores, evidence level and recommendation levels have been detailed (Tables 5–9). The review has been prepared using preferred reporting items for systematic reviews and meta-analyses (PRISMA) guidelines (Moher et al., 2009).

**Table 2:** **Table showing checklist for RCTs according to guidelines recommended by AHRQ**.

Item	Points
Study question – clearly focused?	1
Study population	2
Randomization	2
Blinding	2
Interventions	2
Outcomes	2
Statistical analysis	2
Results	1
Discussion (including limitations and biases)	1
Funding source	2
Total	17
Score	100%

**Table 3:** **Table showing levels of evidence for randomized controlled trials (based on Oxford Center for Evidence-based Medicine)**.

Evidence level	Study design
1	High quality RCTs with narrow confidence intervals
2	Low quality RCTs or high quality cohort studies
3	Case-control studies
4	Case series or poor case-control studies or poor cohort studies or case reports

*High quality RCTs are those having narrow confidence intervals and >80% follow-up rate*.

Low quality RCTS are those with wide confidence intervals, <80% follow-up rate. The Center for Evidence-based Medicine additionally defines low quality cohort studies as “those which have not clearly defined comparison groups and/or failed to measure exposures and outcomes in the same (preferably blinded), objective way in both exposed and non-exposed individuals and/or failed to identify or appropriately control known confounders and/or failed to carry out a sufficiently long and complete follow-up of patients.”

**Table 4:** **Table showing levels of recommendation**.

Term	Level	Evidence levels	Explanation
Recommended	A	1 or 2	Assessment supported by a substantial amount of high quality (levels 1 or 2) evidence and/or based on consensus of clinical judgment
Suggested	B	1 or 2 – few studies 3 or 4 – many studies and expert consensus	Assessment supported by sparse high grade (Level 1 or 2) data or a substantial amount of low grade (level 3 or 4) data and/or clinical consensus
May be considered	C	3 or 4	Assessment is supported by low grade data without the volume to recommend more highly and likely subject to revision with further studies

## Results

**Figure 2:**
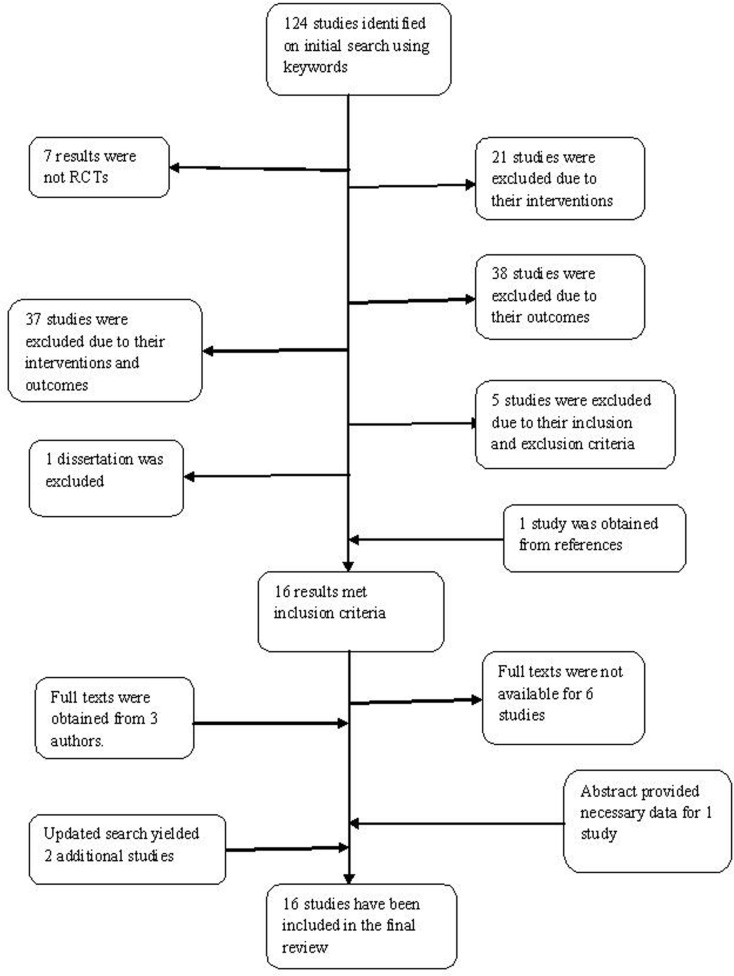
**Literature search**.

Sixteen RCTs met criteria for inclusion in our review. Figure 2 illustrates the process of study extraction.

### Yoga for depression

Four RCTs examining the effects of yoga on depression have been included in this review. Table 5 summarizes each of these studies, including our assessments of their quality.

**Table 5:** **Table showing studies examining yoga for depression**.

Study	Sample	Treatment groups	Intervention	Duration	Outcome measurements	Findings	RCT	Evidence level
Shahidi et al. (2011)	70 depressed women aged 60–80 years from a cultural community in Iran with Geriatric Depression Scale score > 10	Laughter yoga (*n* = 23), exercise therapy (*n* = 23), and wait-list control groups (*n* = 24)	Laughter yoga consisted of brief talk about something delightful, clapping hands, simple chants simulating diaphragmatic breathing, Gibberish sounds. Combines yoga, breathing, and stretching techniques	10 sessions	Yesavage Geriatric Depression Scale and Diener Life Satisfaction Scale (LSS)	Significant improvement in GDS scores in both laughter and exercise groups compared to controls but not when compared to each other	13 (not double blinded, funding information not given)	2 (Low quality RCT due to insufficient follow-up)
Krishnamurthy and Telles (2007)	69 participants (males and females), older than 60, living in a residential home	Stratified sampling and random allocation to yoga, ayurveda, wait-list control groups	Yoga consisted of 7 h 30 min weekly sessions of physical postures, relaxation techniques, regulated breathing, devotional songs, and lectures	24 weeks	Shortened version of Geriatric Depression Scale (GDS)	The yoga group showed significant decrease in depression at 3 and 6 months compared to the ayurveda group	13 (not double blinded, funding information not given)	2 (Low quality RCT due to <80% follow-up rate)
Vedamurthachar et al. (2006)	Males aged 18–55 years with alcohol dependence, admitted for the first time to the de-addiction center of NIMHANS, not having serious medical illnesses, schizophrenia, or mania	SKY – Sudarksha Kriya yoga (*n* = 30) therapy, continued inpatient care (*n* = 30)	SKY consisted of practice of three distinct breathing patterns	2 weeks	BDI scores, ACTH, and cortisol levels	Statistically significant decrease in BDI scores in the SKY group compared to controls. Greater reduction in serum cortisol and ACTH levels in the SKY group	15 (not double blinded)	2 (Low quality RCT due to insufficient follow-up)
Woolery et al. (2004)	28 volunteers aged 18–29 years, with self-reported symptoms of depression, but not on psychotropic treatment and without previous exposure to yoga	Yoga (*n* = 13), wait-list control (*n* = 15)	1 h weekly Iyengar yoga classes, consisting of training in yoga postures	5 weeks	BDI, State-Trait Anxiety Inventory, Profile of mood states, morning cortisol levels	Statistically significant decrease in BDI scores, anxiety scores, and higher morning cortisol levels in the yoga group	13 (not double blinded, funding information not given)	2 (Low quality RCT due to insufficient follow-up)

Shahidi et al. tested 70 elderly women (mean age of 65 years in the intervention groups and 68 years among controls) reporting subjective symptoms of depression with a baseline score of >10 on the Geriatric Depression Scale (GDS) and suggested that 10 sessions of laughter yoga or exercise resulted in significant improvement of depressive symptoms from baseline and compared to a wait-list control group; however the two active treatment groups did not differ from each other (Vedamurthachar et al., 2006). The mild severity makes this study not generalizable to more severe clinical depressives.

In a 24-week study comparing the effects of yoga (7 h weekly) to Ayurveda and wait-list controls among 69 elderly individuals (mean age of 72 years), with self-report of symptoms consistent with depression and baseline mean scores on GDS corresponding to mild illness severity who were not on psychotropic medications, Krishnamurthy et al. reported that in the yoga group, there was a reduction in the scores on the GDS, from the baseline mean score of 10.6 by approximately 20% at 3 months and 40% at 6 months, a change from mild depression to no depression. This was statistically superior to the Ayurveda and wait-list control groups, neither of which demonstrated significant reduction in scores. The main limitations were the potential group interaction benefits of the yoga activity, lack of formal diagnoses according to criteria specified by the Diagnostic and Statistic Manual of Mental Disorders (DSM), relatively modest sample size, and the inclusion of only mildly depressed individuals making it difficult to generalize to more ill patients or to home based yoga (Krishnamurthy and Telles, 2007).

In a study of depression in 60 alcohol dependents males (mean age of approximately 35 years). Vedamurthachar et al. demonstrated that subjects undergoing de-addiction treatment had a statistically significant reduction in their scores on the Beck Depression Inventory (BDI), and concurrent reduction in serum cortisol levels when they received Sudarshan Kriya yoga (SKY) compared to their counterparts receiving routine inpatient care (Vedamurthachar et al., 2006). The BDI scores decreased by 75% at the end of 7 days in the yoga group. The acute alcoholism diagnosis makes it to isolate the effects of yoga versus the effects of alcohol detoxification, and further it was not clear if the subjects met stringent criteria for major depression. While this study documents only a possible acute effect of yoga, it does not provide insights into longer term benefits.

A fourth study focused on treatment naive young adults (mean age of 21.5 years) with self-reported symptoms of depression and scores in the “mild mood disturbance” range on the BDI (Woolery et al., 2004). Woolery et al. found that five weekly sessions of Iyengar Yoga resulted in reduction in scores of depression, from a mean of 12.77 to a mean of 3.90, a value categorized as “normal ups and downs” at the end of 5 weeks, a statistically significant change compared to controls with a score reduction from a mean of 12.07 to 11.0 at the end of the study period. A significant reduction in anxiety and an increase in early morning cortisol level were also reported in the yoga group (Woolery et al., 2004).

None of the studies encountered adverse events in the yoga group though it was not always clear how systematically they were sought for. The drop-out rates were 0% (Vedamurthachar et al., 2006), approximately 27% (Krishnamurthy and Telles, 2007), and 14% (Woolery et al., 2004). Remission and relapse prevention rates have not been determined by currently available studies. Based on our assessment of the available literature according to the RAND/UCLA Appropriateness method, Grade B evidence supporting a potential acute benefit for yoga exists in depression.

### Yoga for schizophrenia

Three RCTs examining the effects of yoga on schizophrenia have been included in this review. Table 6 provides a summary. In a study based at a state psychiatric facility, comprising 18 adult patients (mean age of 37.4 in the yoga group and 48.1 among controls, but without statistical significance in age distribution) diagnosed with schizophrenia or schizoaffective disorder, Visceglia et al. compared the effects of 8 weeks of yoga as an adjunct to anti-psychotic medications with a control group receiving routine inpatient care. The authors reported a reduction in the Positive and Negative Syndrome Scale (PANSS) total score of 25.2 points, from a baseline of 85.1 in the yoga group as well as reductions of 5.9, 6.0, and 13.3 in the positive syndrome, negative syndrome, and General psychopathology sub-scores, all of which were statistically superior to the controls. The secondary outcome measures of physical health and psychological health were significantly improved in the experimental group, as were informal reports of reduced aggression and improved medication compliance. The small sample size, absence of a control intervention, wide range of functionality among participants, and the short duration of follow-up are limitations of this study (Visceglia and Lewis, 2011).

**Table 6:** **Table showing studies examining yoga for Schizophrenia**.

Study	Sample	Treatment groups	Intervention	Duration	Outcome measurements	Findings	AHRQ	Evidence level
Visceglia and Lewis (2011)	Clinically stable patients with schizophrenia, Schizoaffective disorder, with or without PTSD, Axis II pathology admitted to a state psychiatric facility	Yoga (*n* = 10) Wait-list controls (*n* = 8)	Yoga consisted of breathing exercises, warm-ups, and postures, conducted for 45 min twice weekly	8 weeks	PANSS, WHO – quality of life – BREF	Significant improvement in total PANSS, positive syndrome, negative syndrome, general psychopathology. Superior outcomes in physical health and psychological health components of WHO-QOL-BREF	15	2 (Low quality RCT due to limited duration of follow-up)
Behere et al. (2011)	Outpatients with schizophrenia stabilized on anti-psychotics for at least 6 weeks	Yoga (*n* = 34) Exercise (*n* = 31), and Wait-list (*n* = 26)	Yoga module developed by SVYASA consisting of physical postures, breathing exercises, pranayamas. Training for 1 month followed by 2 months of home practice	3 months	PANSS, SOFS, and TRENDS	Significant improvement in positive symptoms, negative symptoms, facial emotion recognition deficits, and socio-occupational functioning in the yoga group in the second and fourth month compared to baseline	15	2 (Low quality RCT since between treatment analysis data not available)
Duraiswamy et al. (2007)	Schizophrenics in the outpatient and inpatient program in aged 18–55 years. Patients were moderately ill, on anti-psychotic medications for months, and on the same drugs for at least 4 weeks	Yoga (*n* = 31), Physical exercise therapy (*n* = 30)	Yoga consisted of asanas, breathing practice, relaxation techniques, and *sithlikarna vyayama*. Training for an hour a day for 3 weeks, followed by continued practice by participants	4 months	PANSS, SOFS 24 (Social and Occupational Functioning Scale, Simpson Angus scale for extra-pyramidal symptoms, AIMS, WHO – quality of life – BREF. Done at baseline and at the end of 4 months	PANSS total and sub-scores, SOFS score reduced significantly in both groups. Statistically significant difference in negative but not positive symptom scores between the yoga and exercise groups	13 (not double blinded, funding information not given)	2 (Low quality RCT since <80% follow-up rate)

*SVYASA, Swami Vivekananda Yoga Anusandhana Samsthana; PANSS, Positive and Negative Syndrome Scale; SOFS, Socio-Occupational Functioning Scale; TRENDS, Tool for Recognition of Emotions in Neuropsychiatric Disorders*.

Behere et al. compared the adjunctive effects of yoga with exercise wait-list controls in their 3 month study of 91 anti-psychotic stabilized adult outpatients with schizophrenia with baseline Clinical Global Impression (CGI) score less than or equal to 3. The authors reported reduction in PANSS positive and negative symptom scores by 17 and 20%, respectively, statistically superior to the other two groups, as well as significant improvements in facial emotion recognition deficits, and socio-occupational functioning. Significantly higher baseline scores in the PANSS negative sub-scale and facial emotional recognition deficit in the yoga group, variation in the amount of yoga practice at home during the last 2 months of the study, limited follow-up are drawbacks of this study, and the inclusion of stable outpatients limit its generalizability to more severely ill individuals (Behere et al., 2011).

In a study of 61 anti-psychotic stabilized (mean dose of around 470 mg/day in Chlorpromazine equivalents) inpatients and outpatients (mean age around 32 years) with schizophrenia (CGI illness severity score of 4.8 and 5.2 in the yoga and control groups) Duraiswamy et al. compared the effects of yoga with exercise, as adjuncts to anti-psychotic medications. Participants were taught yoga and exercise for 3 weeks, followed by encouragement of continued practice with monitoring of adherence. The authors reported a reduction in the total PANSS score by 25.09 points, corresponding to a moderate-to-large effect size of 0.74 in the yoga group, a greater reduction in the negative sub-scale (7.71 points, from a baseline of 21.9), but no statistically significant change between the two groups in the positive sub-scale. The yoga group demonstrated an improvement of socio-occupational functioning, with an effect size of 0.48 in the Socio-Occupational Functioning Scale (SOFS). Notable limitations of the study include its modest sample size and unclear assessment of continued home practice of the interventions (Duraiswamy et al., 2007).

There were no adverse events, attributable to yoga reported in any of the studies, although it is not clear how this assessment had been performed. It is difficult to separate the effects of yoga from the benefits of group interaction. Assessments of change in the dose of anti-psychotics, relapse rates, and hospitalization rates have not been performed in any of the existing studies. Based on our assessment of the available literature according to the RAND/UCLA Appropriateness method, Grade B evidence supporting a potential benefit for yoga as an adjunct to anti-psychotic treatment in chronic schizophrenia.

### Yoga for Attention-Deficit Hyperactivity Disorder

Two RCTs examining the effects of yoga on Attention-Deficit Hyperactivity Disorder (ADHD) have been included in this review (Table 7). In a cross-over study of 19 children with mean age around 10 years, diagnosed with ADHD meeting both International Classification of Diseases-10 (ICD-10) and Diagnostic and Statistical Manual of Mental Disorders-IV (DSM-IV) criteria (which included children with attention disorders, hyperkinetic disorder of social behavior, and not otherwise specified hyperkinetic disorder). Haffner et al. compared the effects of yoga with “conventional motor exercises,” comprising of well known active games as adjuncts to pharmacotherapy for 34 weeks. The authors report superior efficacy of yoga with effect sizes of 0.77, 0.71, 0.60, and 0.97 in the total scale, attention-deficit sub-scale, hyperactivity sub-scale, and impulsiveness sub-scale, respectively, of a German ADHD rating scale for parents and teachers. They also found a significant sequence effect on the Dartmond Attention Test (DAT), such that the group which performed yoga followed by motor exercises showed a higher improvement in scores after yoga but their mean score change at the end of the study was lower than the other group, which according to the authors may indicate that the performance gain after yoga was lost after the conventional motor exercise intervention (Haffner et al., 2006). The modest sample size, carry-over effects from the cross-over design, limited follow-up, and exclusion of children with severe behavioral symptoms which are frequently co-morbid with ADHD are limitations of the study.

**Table 7:** **Table showing studies examining yoga for ADHD**.

Study	Sample	Treatment groups	Intervention	Duration	Outcome measurements	Findings	RCT score	Evidence level
Haffner et al. (2006)	19 children diagnosed with ADHD, with the exclusion of those with severe developmental disabilities, IQ < 70, and severe behavioral disturbances	Yoga and a control group consisting of conventional motor exercises. Cross-over design (YE and EY). Subjects were continued on their medications or complementary therapy	Two hourly sessions of Hatha yoga per week for 8 weeks, followed by a 6-week training break and 8 weeks of conventional motor exercises	34 weeks	Parent, teacher ratings of ADHD (FBB-HKS) test scores on an attention task (DAT). Measurements done before an intervention, between interventions, and after the second intervention	Yoga was superior to conventional training with effect sizes between 0.60 and 0.97. Treatment more effective in children on medications	13 (not double blinded, funding information not given)	2
Jensen and Kenny (2004)	16 boys diagnosed with ADHD according to DSM-IV criteria and on medications. Included children with co-morbid anxiety and learning disorders but excluded those with previous diagnoses of Oppositional defiant disorder and Conduct Disorder	Yoga group (*n* = 11), Control group consisting of co-operative activities (*n* = 8). Cross-over design	20 weekly yoga sessions lasting for an hour each. Yoga consisted of respiratory training, postural training, relaxation training, and concentration training (*tratak)*	20 weeks	Conners Parent and Teacher Rating Scales. (CPRS and CTRS)	Yoga group showed significant improvement on five sub-scales of CPRS (Oppositional, Global Index total, Global Index emotional lability, and Global Index Restless/Impulsive, ADHD Index) Control group showed improvement on three different sub-scales (Hyperactivity, Anxious/shy, and Social problems) Both groups improved significantly on CPRS perfectionism, DSM-IV hyperactive/impulsive, and DSM-IV total. No significant change on CTRS	13 (not double blinded, funding information not given)	2

*DAT, Dortmund Attention test; YE, yoga followed by exercise; EY, exercise followed by yoga; TOVA, Tests of Variables of Attention*.

Jensen et al. compared the effects of yoga with a control group comprising of games incorporating talking, listening, and sharing equipment for 20 weeks in their cross-over study of 16 children (mean age of 10.63 and 9.35 years in the yoga and control groups), diagnosed with ADHD according to DSM-IV criteria and continued on pharmacotherapy. They reported significant post-intervention improvement in scores on the Conners’ Parent Rating Scales (CPRS), namely the Oppositional (Cohen’s *d* of 0.77), Global index Emotional lability (Cohen’s *d* of 0.79), Global Index Total (Cohen’s *d* of 0.73), Global Index Restless/Impulsive (Cohen’s *d* of 0.73), ADHD index (Cohen’s *d* of 0.29), and Perfectionism (Cohen’s *d* of 0.58) sub-scales but not in the Hyperactivity, anxious/shy, and social problems sub-scales, where the controls fared better. It is notable that neither group showed statistically significant improvement in scores rated by teachers, and the authors have suggested that this result may be obscured by the fact that assessments in schools occur when children are medicated, while that by parents is during unmedicated times. There were anecdotal reports by parents, of improved homework compliance and yoga being an effective calming technique during episodes or behavioral escalation. The limited follow-up limits the understanding of the maintenance effects of yoga (Jensen and Kenny, 2004).

Neither study has reported adverse events in the yoga group, although it is not clear how side effect assessment was performed. Details of pharmacotherapy for ADHD, change in dose during the course of the study have not been provided. Based on our assessment of the available literature according to the RAND/UCLA Appropriateness method, Grade B evidence supporting a potential benefit for yoga as an adjunct to pharmacotherapy in ADHD in children.

### Yoga for eating disorders

Two RCTs examining the effects of yoga on eating disorders have been included (Table 8). McIver et al. included 90 overweight or obese women (mean age of 40.1 and 42 years in the yoga and control groups) with self-reported symptoms of binge eating (listed in DSM-IV TR appendix) and a mean Binge Eating Scale (BES) score of around 28, corresponding to severe binge eating. They reported that BES score decreased by approximately 50% after 12 weeks of yoga, corresponding to an improvement from “severe” binge eating to the “absence” of binge eating, statistically superior to wait-list controls who did not demonstrate any improvement. The authors also report a lower attrition rate in the yoga group (26%) compared to controls (32%) and an increase in overall physical activity, measured by the International Physical Activity Questionnaire (IPAQ). Limitations of this study include the procurement of data by self-report, absence of details of concurrent pharmacotherapy in the paper, and difficulty separating the true effects of yoga from that of increased contact and attention received by the yoga group during the course of the study. It would have been useful to assess whether participants perceived yoga as a way of losing weight or for overall mental health (McIver et al., 2009).

**Table 8:** **Table showing studies examining yoga for eating disorders**.

Study	Sample	Treatment groups	Intervention	Duration	Outcome measurements	Findings	RCT	Evidence level
McIver et al. (2009)	90 women aged 25–63 from a community meeting criteria for Binge eating disorder, BMI > 25	Yoga (*n* = 45), controls (*n* = 45)	60 min weekly sessions (pranayama + hatha yoga + nidra yoga)	12 weeks	Primary – BES Secondary – IPAQ BMI, hips, and waist measures	Statistically significant reductions in binge eating and increase in physical activity in the yoga group	13 (not double blinded, funding information not given)	2 (<80% Follow-up)
Mitchell et al. (2007)	113 women who responded to advertisements calling for women dissatisfied with their bodies	Cognitive dissonance (*n* = 30), yoga (*n* = 33), or control (*n* = 30) groups	Weekly for 45 min	6 weeks	EDDS, BES, STAI, CES-D, EDI, IBSS-R, TFEQ, TAS-20, and BSQ-R-10	No differences between the yoga and control groups. Significant improvements in the dissonance groups on the ED-BD, ED-DFT, EDDS, BSQ-R-10, STAI, and TAS	15 (not double blinded)	2

*EDDS, Eating Disorder Diagnostic Scale; BES, Binge Eating Scale; STAI, State-Trait Anxiety Inventory; CES-D, Center for Epidemiological Studies Depression Scale; EDI, Eating Disorder Inventory; IBSS-R, Ideal Body Stereotype Scale-Revised; TFEQ, Three Factor Eating Questionnaire; TAS-20, Toronto Alexithymia Scale; BSQ-R-10, Body Shape Questionnaire-Revised-10; IPAQ, International Physical Activity Questionnaire*.

The second study in this group included 113 women (mean age of 19.56 years) reportedly “dissatisfied with their bodies,” recording mean baseline scores on the Eating Disorder Diagnostic Scale (EDDS) of 26.34, 30, and 22.55 in the yoga, cognitive dissonance therapy, and wait-list control groups, respectively, where a score > 16.5 is strongly suggestive of illness. The authors used a number of outcome measurements (see Table 9), which include assessments of eating disorder, binge eating, body shape perception, alexithymia, anxiety, and depression to compare the three groups for a duration of 6 weeks. This study reported significant improvement in the group which received therapy based on cognitive dissonance, but not in the yoga or wait-list control groups (Mitchell et al., 2007). Since only one study yielded positive results, we did not grade the evidence for this category.

**Table 9:** **Table showing studies examining yoga for sleep complaints**.

Study	Sample	Treatment groups	Intervention	Duration	Outcome measurements	Findings	RCT	Evidence level
Chen et al. (2009)	Community-dwelling, ambulatory, adults of mean age of 69.2 years, without previous training in yoga, cognitively alert, and independent or mildly dependent in self-care	Silver yoga (*n* = 62), Control group (*n* = 66)	Silver yoga exercises lasting for 70 min, conducted three times a week. Consisted of warm-up, postures, hatha yoga, relaxation, and guided imagery meditation	6 months	PSQ1 (Chinese version), TDQ (Taiwanese Depression Questionnaire), SF-12 health survey, and (Chinese version)	At 3 and 6 months, significantly better scores on PSQI and less depression were found in the yoga group compared to baseline and compared to controls	15 (not double blinded)	2 (Low quality RCT since the SD was large)
Manjunath and Telles (2005)	69 residents from a home for the aged, stratified on the basis of age	Yoga (*n* = 23), ayurveda (*n* = 23), and wait-list control (*n* = 23) group	Yoga consisted of physical postures, relaxation techniques, regulated breathing, and exercises on yogic philosophy	6 months	Sleep latency, duration, awakenings, feeling of being rested, and day-time napping. Assessed at baseline, 3, and 6 months	Yoga group showed a significant decrease in sleep latency, increase in sleep duration compared to baseline. Between treatment effects were not significant	15 (not double blinded)	2 (Low quality RCT due <80% follow-up rate)
Cohen et al. (2004)	39 adult patients with lymphoma who were undergoing or had completed treatment in the past 12 months	Tibetan Yoga (*n* = 20), wait-list controls (*n* = 19)	Tibetan Yoga consisted of controlled breathing, visualization, mindfulness, and postures	7 yoga sessions	PSQI, Impact of Events Scale, STATE, CES-D, and Brief Fatigue Inventory	Tibetan yoga group showed statistically significant improvement in sleep latency duration, quality, and the total score, but none of the other outcomes	13 (not double blinded, funding information not given)	2 (Low quality RCT due to insufficient follow-up)

*PSQI, Pittsburgh Sleep Quality Index*.

### Yoga for sleep complaints

Three RCTs examining the effects of yoga on sleep complaints have been included in this review (Table 9). In their study of 139 ambulatory, community-dwelling, elderly (mean age of 69.2), cognitively able participants without previous training in yoga. Chen et al. compared the effects of yoga (three times a week for 6 months) to wait-list controls. The mean baseline total Pittsburgh Sleep Quality Index (PSQI) scores were 4.65 and 5.47, respectively, for yoga and control groups, where total score > 5 is associated with poor sleep quality. The authors report a reduction in the total PSQI score, through 4.48 at 3 months to 3.34 at 6 months in the yoga group, statistically superior to controls who demonstrated an increase in the score, implying poor outcome. The yoga group also demonstrated statistically superior outcomes related to sleep latency, day-time dysfunction, secondary outcomes of depression, physical, and mental health perception, all of which reportedly worsened among controls. The reliance on self-report for inclusion in the study, absence of formal DSM diagnoses of either primary or secondary insomnia, baseline mean score outside of the range considered “poor sleep,” absence of information about use of sleep aids limit the generalizability of these findings (Chen et al., 2009).

In a study conducted at a home for the aged in India, Manjunath et al. compared the effects of 6 months of training in yoga versus an ayurvedic preparation on 69 elderly subjects (mean age of 70.1, 72.1, and 72.3 in the yoga, ayurveda, and wait-list control groups) with self-report of sleep impairment, but the absence of formal diagnosis of a sleep disorder at baseline. The authors reported a mean reduction in sleep latency of approximately 10 min and an increase in duration of approximately 60 min in the yoga group, a significant finding compared to the two control groups, neither of whom demonstrated comparable improvement. Of note, the sleep latency was fairly high at 25.83 min in the yoga group, even at the end of the study. The modest sample size, absence of formal DSM diagnoses, the presence of statistical significance within treatments but not between treatments for sleep latency are notable limitations (Manjunath and Telles, 2005).

Cohen et al. examined the effects of seven weekly sessions of Tibetan yoga (which combined training in breathing, relaxation, and postures with guided imagery), comparing it to wait-list controls on 39 adults (mean age of 51 years) with lymphoma who were either receiving chemotherapy or had received it within the past 1 year. Participants reported subjective sleep impairment and recorded baseline PSQI scores of 6.5 and 7.2, respectively, in the yoga and control groups, corresponding to “poor sleep quality” according to scoring guidelines. Formal DSM diagnoses of insomnia had not been established. The yoga group demonstrated a statistically superior reduction in the total PSQI score, a reduction from a mean of 6.5 to 5.8, compared to controls who recorded a mean score of 8.1 at the end of the study. Scores of sleep quality (improved from 0.90 to 0.85), latency (improved from 1.10 to 0.75), and duration (improved from 0.85 to 0.89) were favorable in the yoga group, whereas the controls did poorly on all of the above parameters. The yoga group, but not controls showed a statistically significant reduction in the need for sleep aids – details of agents used and doses have not been specified. While there was improvement in sleep related parameters, depression, and state anxiety did not change. The modest sample size, unclear distinction between primary sleep disorders and those secondary to a mood, or anxiety disorder are drawbacks of this study (Cohen et al., 2004).

None of the studies reported adverse effects attributable to yoga, although it is not clear how they were assessed. Based on our assessment of the available literature according to the RAND/UCLA Appropriateness method, Grade C evidence supporting a potential benefit for yoga exists for sleep complaints.

### Yoga for cognition or conditions influencing cognition

Two studies have been included in this review, the details of which can be found on Table 10. Sharma et al. examined the adjunctive effects of 8 weeks of yoga on neurocognitive functions in 30 adults (age range between 18 and 45) meeting criteria for Major Depression, comparing it to a group which received only medications. Outcomes included measures of working memory, executive function, visual attention, task switching ability, and visual scanning, as outlined in Table 10. The authors report that while both groups demonstrate improvement in the Letter Cancelation Test (LCT), a measure of attention, concentration, and visuospatial function), Trail making tests A and B (measures of visual attention and task switching), the yoga group demonstrated superior results in LCT, and separated from the controls in the Reverse digit span test assessing short-term memory (Sharma et al., 2006). The modest sample size, lack of information about the severity of depression in the abstract are limitations and it is unclear if the improvement in these measures of cognition in the yoga group are a direct effect on cognition or secondary to greater improvement in depression mediated through yoga.

**Table 10:** **Table showing studies examining yoga for cognition**.

Study	Sample	Treatment groups	Intervention	Duration	Outcome measurements	Findings	RCT score	Evidence level
Sharma et al. (2006)	30 individuals aged 18–55 years with MDD, on anti-depressants	Sahaja yoga + medications (*n* = 15) only medications (*n* = 15)	Details not specified	8 weeks	Neurocognitive tests (LCT, TTA, TTB, RFFT, FDS, and RDS)	Significant improvement in LCT, TTA, TTB in both groups. Greater improvement in LCT in yoga group. Significant improvement in RDS scores only in yoga group	Abstract	Not assessed since full text was not available
Oken et al. (2006)	135 men and women aged 65–85 years. Excluded patients with severe medical problems, alcoholism, and drug dependence. Baseline level of cognitive function not specified	Hatha yoga (*n* = 44), walking (*n* = 47), and wait-list controls (*n* = 44)	Iyengar yoga postures, classes were conducted for 90 min every week along with home practice. Progressive relaxation, visualization, and meditation techniques were introduced	6 months	Stroop color and word tests, quantitative EEG measure of alertness (posterior median frequency)	No significant difference in measures of cognition	15 (not double blinded)	2 (results not statistically significant)

*LCT, Letter Cancelation Test; TTA, Trail Making Test “A”; TTB, Trail Making Test “B”; RFFT, Ruff Figural Fluency Test; FDS, forward digital span; RDS, reverse digital span*.

In a study of 135 elderly individuals (mean age of 71.5, 73.6, and 71.2 in the yoga, exercise, and wait-list groups, respectively). Oken et al. compared the effects of yoga with exercise and wait-list controls over 6 months, focusing on measures of alertness using EEG and the Stroop Color and word tests. Baseline cognitive assessments are not reported to have been performed; individuals with severe medical illnesses were excluded, as were those with experience in yoga over the last 6 months. Significant changes in measures of cognition were not demonstrated in any of the groups. The yoga group did demonstrate improvement in quality of life measures related to a sense of well-being as physical measures such as forward flexibility and timed one leg standing. The negative results notwithstanding, the absence of specification of baseline cognitive status is a drawback of this study (Oken et al., 2006).

## Discussion

### Key findings

To our knowledge, this is the first review to systematically examine the published literature on benefits of yoga for several major psychiatric illnesses. Based on our assessment of the available literature according to the RAND/UCLA Appropriateness method, Grade B evidence supporting a potential acute benefit for yoga exists in depression (four RCTs), as an adjunct to medications in Schizophrenia (three RCTs) and ADHD (two RCTs), and Grade C evidence supports the benefit of yoga for sleep complaints (three RCTs).

Studies have found reasonable benefit in mild depression, even in the absence of pharmacotherapy. Studies of yoga in schizophrenia have yielded evidence of benefit as an adjunct to medications in improving positive and negative symptoms, quality of life, and socio-occupational functioning. The RCTs examining yoga in ADHD have demonstrated moderate-large effect sizes, comparable according to the authors, to other alternative therapies such as biofeedback and relaxation in ADHD (Haffner et al., 2006). Three RCTs suggest substantial benefit for sleep complaints, although the absence of formal DSM diagnoses in these studies is limiting. RCTs in cognitive disorders and eating disorders yielded conflicting results. Of note, Grade B implies that the assessment is supported by sparse high grade data or a substantial amount of low grade data and/or clinical consensus and Grade C suggests that the assessment is supported by low grade data without the volume to recommend more highly and likely subject to revision with further studies (Fitch et al., 2000).

### Limitations based on search strategy and inclusion/exclusion criteria

Although yoga has been used as a treatment for a wide variety of psychiatric conditions and distress, we have focused on the major broad categories of psychiatric disorders, namely depression, schizophrenia, eating disorders, ADHD, sleep complaints, and cognitive impairments. We excluded studies on sub-threshold symptoms such as coping, general well-being as well as studies conducted on individuals without psychiatric diagnosis. This was done to minimize the possibility that observed effects are merely a reactive change to a new event in normal individuals. Our search term “anxiety” yielded studies on post-traumatic stress, state, and trait anxiety but these studies had specifically excluded individuals with pre-established psychiatric diagnoses and hence did not make it to the final review.

### Limitations based on study methodology

Few studies have provided details on how randomization had been performed. Studies included in our review consist of various sub-types of yoga and the description of the intensity of yoga has not been specified in many studies. The number of studies for each sub-type of yoga is very small, therefore, for the purpose of our review, which is the first of its kind, we considered sub-types which included similar basic components, namely controlled breathing, relaxation, and postural training to be equivalent. Due to the nature of the intervention, blinding of subjects is challenging, while information regarding blinding of the assessor has not been provided in most studies. Analogous to other interventions such as exercise, where they may be effects of group intervention, it is difficult to isolate benefits of being in a group from that derived from yoga alone in our studies. This may be particularly the case in studies with wait-list controls, where it is difficult to establish if the observed changes are due to the effect of yoga or merely expectation. The sample sizes are small in many studies and the generalizability of benefits noted in participants who demonstrate the motivation to participate and comply in studies of yoga may be questionable. The severity of illness has varied across studies, and it is of concern if the findings from results of mildly ill individuals (such as the depression studies) can be extrapolated to those with severe illness. Although adverse effects have not been reported in these studies, details of how the assessment had been done are lacking.

## Conclusion

Our systematic review finds emerging scientific evidence to support a role for yoga in treating depression, sleep complaints consistent with both popular beliefs and biological studies, and having adjunctive value in schizophrenia and ADHD. The evidence in other disorders remains less well established. Given the growing popularity of yoga, it would be important for the field to attempt to replicate and extend these findings in larger, multi-center, randomized, blinded (at least single blinded) studies with the control group receiving alternative treatments, preferably using Good clinical practice (GCP) guidelines. Biomarker research, such as through functional magnetic resonance imaging (MRI) and Positron Emission Tomography (PET) studies, and molecular markers (genomics, metabolomics, and proteomics), would facilitate greater scientific understanding at a neurobiological level, of this 5000-year-old revered practice.

## Conflict of Interest Statement

P. Murali Doraiswamy has received research grants and/or advisory fees/honoraria from several government agencies, media, and pharmaceutical companies. He owns stock in Sonexa and Clarimedix.
